# Exploring the Effects of Manual Therapy on Somatosensory Tinnitus and Dizziness: A Randomized Controlled Trial

**DOI:** 10.3390/jcm14134579

**Published:** 2025-06-27

**Authors:** Andrea Bökel, Andreas Fobbe, Anke Lesinski-Schiedat, Christian Sturm

**Affiliations:** 1 Department of Rehabilitation and Sports Medicine, Hannover Medical School, 30625 Hanover, Germany; fobbe.andreas@mh-hannover.de (A.F.);; 2 Department of Otorhinolaryngology, Hannover Medical School, 30625 Hanover, Germany

**Keywords:** manual therapy, neck muscle, tinnitus, dizziness

## Abstract

**Objectives**: Muscular dysfunction of the cervical spine is the most likely cause of somatosensory tinnitus and dizziness. Some patients can modulate their tinnitus through movement or palpation. This study aimed to investigate the effect of manual therapy on muscle pressure pain, range of motion in the cervical spine, and tinnitus and dizziness. **Methods**: A pilot randomized controlled trial with a waiting-group design was conducted in a university hospital setting. Participants in the intervention group received manual therapy to the head and neck region combined with stretching exercises and muscle relaxation techniques such as releasing tense muscles and myofascial trigger point therapy involving muscle and connective tissue techniques. The primary and secondary outcome measures were pressure pain, tinnitus modulation by head and neck muscles, and range of motion of the cervical spine. Tinnitus and dizziness were assessed before and after the intervention using the Tinnitus Handicap Inventory and the Dizziness Handicap Inventory. **Results**: After the intervention, significant differences were observed in pressure pain, tinnitus modulation, and range of motion as well as the Tinnitus Handicap Inventory (U = 644; *p* < 0.001) and the Dizziness Handicap Inventory (U = 133.5; *p* = 0.010), favoring the intervention group. **Conclusions**: The results demonstrate that manual therapy significantly altered pressure pain in the head and neck muscles as well as symptoms such as tinnitus and dizziness in the intervention group. Manual therapy may be beneficial in treating cervicogenic somatosensory tinnitus, provided that other potential causes such as otorhinolaryngological pathology have been ruled out.

## 1. Introduction

Tinnitus affects more than 740 million people worldwide. Furthermore, more than 120 million people consider it to be a major problem [1]. Tinnitus is the perception of sound in the absence of obvious acoustic stimuli. It is categorized according to whether the perceived sound has an identifiable source (objective tinnitus) or not (subjective tinnitus). Objective tinnitus can be caused by muscle contractions or blood vessels, for example. Subjective tinnitus is by far the most common type of tinnitus [2]. Further definitions include primary tinnitus, which is idiopathic and not necessarily associated with sensorineural hearing loss, and secondary tinnitus, which is associated with a specific cause [3]. The causes can be located in the inner ear (e.g., from vestibular schwannoma or Ménière’s disease), in the middle ear (e.g., otosclerosis or otitis media), or the external ear (e.g., cerumen impaction or otitis externa). Alternatively, it may have non-auditory causes such as vascular anomalies or myoclonus or nasopharyngeal carcinoma [3]. Subjective tinnitus has also been associated with elevated HbA1c and triglyceride levels, while chronic tinnitus might be influenced by elevated glucose levels [4]. When patients are not affected by tinnitus, it is called compensated tinnitus. This is divided into grade 1 (no distress) and grade 2 (occurring in silence and causing distress). If patients are suffering from tinnitus, it is called decompensated tinnitus. This inevitably leads to permanent private and/or occupational impairment (grade 3) or complete decompensation, including inability to work (grade 4). Decompensated tinnitus is often accompanied by other conditions such as fatigue, sleep disturbances, and depression and anxiety disorders [5,6]. Quality of life is negatively correlated with the severity of tinnitus-related distress [7,8]. This distress also significantly influences the appearance of depressive symptoms [9]. This reflects the importance of effective therapy.

Cervicogenic somatosensory tinnitus (CST) is a subgroup among the various causes of tinnitus. It is caused and triggered by dysfunctions of the cervical spine, temporomandibular joints, and musculoskeletal structures of the head and neck region. Somatosensory tinnitus typically changes in terms of loudness and frequency in response to movement and stimulation of muscles and joints [6]. Since CST was first identified as a subtype of subjective tinnitus, numerous studies have provided neurophysiological insights into this condition. Attempts have also been made to define standardized diagnostic criteria, but these can never be conclusive or unambiguous [10]. Levine (1999) [11] reported the somatic modulation of tinnitus. Patients demonstrated the ability to influence their tinnitus by applying tension and movement maneuvers to the cervical spine and head. In some cases, the pathological ringing in the ears became louder, while in others it became quieter [11]. Recent studies have also investigated the influence of manual therapy on tinnitus and found positive effects.

Biesinger emphasized the importance of cervical dysfunction in otorhinolaryngology, highlighting the need for precise functional diagnostics, particularly with regard to the upper cervical spine [12]. It has been suggested that dizziness may be caused by a neck condition because it is associated with the rotation of the head and cervical spine [13,14]. The primary diagnostic methods are medical imaging and objective vestibular testing, which establish whether a patient’s dizziness is caused by an underlying neck condition. However, the causes and physiology of the relationship between muscles, tinnitus, and dizziness symptoms are not well understood.

Another hypothesis regarding this relationship is the brainstem irritation model. This model states that increased electrical stimuli from tense muscles cause misconnections in cranial nerve nuclei, triggering “short circuits” that activate the cochlear and vestibular nuclei [15]. Furthermore, neuroanatomical studies in animals have conclusively demonstrated the presence of muscular somatic projections from the upper cervical spine to the cochlear nuclei [16,17]. This stimulus state (irritation) is probably triggered by a large number of somatic afferents arriving in the brainstem. These afferents originate from the trigeminal nerve area on the one hand and from the upper three cervical vertebral segments (C1–C3). The two neck extensor muscles, the splenius capitis and the semispinalis capitis muscles, are part of the autochthonous back muscles and are innervated by the posterior rami of the spinal nerves. The splenius capitis muscle arises from segments C2–C3 (splenius capitis muscle) and C3 (semispinalis capitis muscle) [18]. Dysfunction in this area, such as muscle tension, undoubtedly triggers the aforementioned irritable state, causing the afferents to converge on the posterior cochlear nucleus in the brainstem. This establishes a link between “muscular information” and tinnitus, which the patient perceives as non-auditory. Currently, there is no scientific evidence to support this theory and it is also difficult to obtain due to the fragility of brainstem tissue, which does not appear to be suitable for electrophysiological measurements.

Recent studies have described the correlation between temporomandibular dysfunction and tinnitus [19]. Some studies have documented the efficacy of physiotherapeutic interventions targeting the cervical spine and jaw in alleviating tinnitus symptoms [20,21]. Other results of this study have been published in a German-language journal [22].

This article investigates the effects of manual therapy on (i) the muscles of the head and neck region, assessed by pressure pain and tinnitus modulation, and (ii) the range of motion of the cervical spine as well as (iii) the difficulties experienced by participants due to tinnitus and dizziness.

## 2. Materials and Methods

We conducted a prospective randomized controlled trial with a waiting-group design. Participants were eligible if they were aged 18 or over and had acute or chronic subjective tinnitus with or without dizziness and/or concomitant cervical spine complaints. They also had to exhibit modulation of tinnitus and/or dizziness in response to manual muscle stimulation [10].

The criteria for tinnitus modulation are as follows [10]:

1. The patient can alter their tinnitus by voluntarily moving their head, neck, jaw, or eyes.
2. The patient can change their tinnitus through specific somatic maneuvers.
3. Tinnitus can be influenced by applying pressure to myofascial trigger points.

Patients were excluded if they had objective tinnitus, had undergone previous neck or ear surgery, had known cranial or cervical vascular anomalies, had severe cognitive impairment, had experienced an acute injury, or had received manual therapy to the head or neck in the last eight weeks prior to entering the study.

Patient were recruited from the outpatient departments of otorhinolaryngology and rehabilitation and sports medicine at a university hospital through a public call on the hospital’s website and by approaching patients during consultation hours. Some participants were referred to the study by otolaryngologists, while others chose to participate in the study on their own without prior diagnostic testing. During recruitment, a clinical examination was performed to assess symptoms of tinnitus or dizziness. During the initial consultation, we did not systematically differentiate between primary and secondary tinnitus.

As recommended in the German tinnitus guidelines [23], the modulability of symptoms was tested during an initial cervical spine examination involving stimulation of the relevant muscles. Once modulability had been established, somatosensory tinnitus was suspected. In these cases, no further diagnostics were initiated, and the manual therapy intervention was started in the intervention group. Where there was no significant improvement in symptoms, further diagnostics by relevant specialists were recommended.

The first patient was enrolled on 23 June 2020 and the last on 28 October 2020.

**Figure 1:**
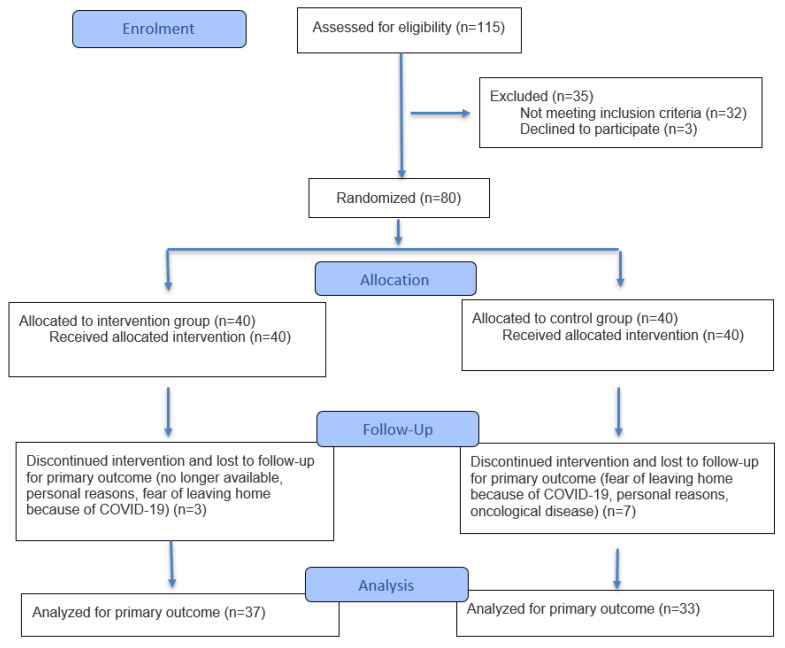
CONSORT flow diagram (*n*: number of participants) according to Hopewell et al. [25].

Figure 1 shows the patient flow. The study was designed as a waitlist group trial. Patients in the intervention group (IG) underwent an examination (T0) and completed an assessment, including THI and DHI, to document their individual complaints. This was promptly followed by a series of manual therapy sessions. A second examination (T1) was carried out, with the assessments, including THI, DHI, and manual medical examinations, being repeated one to two weeks after the intervention. The control group (CG) underwent the same initial examination (T0) as the IG. A second examination was performed after six weeks (T1) to assess whether there had been any spontaneous improvement in symptoms, as shown in Figure 1. The standardized examination was performed by an orthopedic specialist.

In accordance with ethical considerations, the CG also received a series of manual therapies after the T1 examination, as did the IG.

Manual therapy (MT) was provided at the Outpatient Physiotherapy Department of Hannover Medical School and at three specialized private physiotherapy practices in Hannover. All of the physiotherapists involved had completed two years of certified training in manual therapy. They were informed about the procedure and the aims of the study before the study began. The manual therapy combined stretching exercises with muscle relaxation techniques such as releasing tense muscles and myofascial trigger point therapy with muscle and connective tissue techniques [24]. The therapists demonstrated and explained the stretching exercises to the patients. Patients were instructed to perform these exercises at home for approximately 15 min each day. The waitlist group received no treatment.

At the beginning of the study, all patients were fully informed about the procedure, the study’s objectives, and the examination methods being used. An information sheet was distributed and verbally explained. The standardized examination was performed by an orthopedic specialist. Written informed consent to participate in the study was obtained from each patient.

A similarly designed study was used as a reference to plan and calculate the sample size [21]. The calculation was based on a power of 80% and an effect size of d = 0.7. This ensured that changes in the outcome parameters could be detected (α = 0.05) in the analysis, with two repeated measures and two study groups. The analysis sample required 34 patients per study group. Allowing for a dropout rate of 15%, 40 patients were recruited per group, totaling 80 patients. The G*Power 3 program (Version 3.1.9.7.) was used for the calculation.

### 2.1. Measurements

The muscles were manually examined according to the standards of Travell and Simons [24]. The following muscles were examined in detail for pressure pain and modulation of tinnitus and dizziness: the splenius capitis, the semispinalis capitis, the trapezius pars descendens, the temporalis, levator scapulae, the floor-of-mouth muscles, the masseter, the sternocleidomastoideus, and the pterygoideus medialis muscles.

The modulation of tinnitus and dizziness via the respective painful pressure point of the muscle were manually tested, as was pressure pain. Modulation of tinnitus or dizziness was defined as the ability to alter the sensation of tinnitus or dizziness to a positive or negative state. The examiner stimulated a painful area (trigger point) in the muscle under manual examination with their finger, and the patient indicated if they perceived a change. In the case of tinnitus modulation, this could manifest as a change in frequency, quality, or volume. Additionally, the noise could disappear completely for a short time. For dizziness, this would be an increase or a decrease. The quality might also change. For example, the feeling of dizziness could change to spinning or staggering dizziness. Participants were encouraged to report any changes during the examination. The dichotomous rating scale resulted in two definitive answer options, “Yes” (any change, even slight) and “No” (no change).

Cervical spine mobility was measured using a CROM-3 device (manufactured by Performance Affizing Associates Inc., St. Paul, MN, USA) [25]. This plastic frame is worn like a pair of glasses and allows for the precise determination of cervical spine mobility. The measurable parameters are lateral inclination, rotation and inclination, and reclination. The CROM-3 device has been shown to be reliable and valid [26,27]. Values in degrees were documented using a self-administered examination form.

### 2.2. Tinnitus Handicap Inventory (THI) Questionnaire

The THI assesses the nature and extent of any difficulties that respondents might experience as a result of their tinnitus. Consisting of 25 questions, the THI is divided into the following three subscales: the catastrophic scale, the functional scale, and the emotional scale. The response options are as follows: Yes = 4 points, Sometimes = 2 points, and No = 0 points. Participants can score a maximum of 100 points, and the severity scale is as follows: Grade 1 = light (0–16), Grade 2 = mild (18–36), Grade 3 = moderate (38–56), Grade 4 = severe (58–76), and Grade 5 = catastrophic (78–100) [28]. The German version is reliable for clinical studies [29]. A reduction of seven points represents a clinically relevant improvement. A reduction of 17 points represents a major improvement [30].

### 2.3. Dizziness Handicap Inventory (DHI)

This questionnaire was developed to assess the problems experienced by patients as a result of vestibular or balance disorders. The DHI comprises 25 questions, which are divided into the following three subscales: physical, functional, and emotional. The response options are Yes = 4 points, Sometimes = 2 points, and No = 0 points. The severity scale is Mild (0–30), Moderate (31–60), and Severe (61–100). The German version is reliable for measuring dizziness. A change in the German version score of at least nine points is required to indicate improvement or worsening [29].

### 2.4. Randomization Procedure

For randomization we marked 40 cards with an ‘A’ (for IG) and 40 cards with a ‘B’ (for CG), placing them in 80 identical opaque envelopes. The envelopes were distributed by an administrative staff member who was not involved in the medical management of the study. Group allocation was thus ascertained.

The physiotherapists were blinded to the group allocation of the patients. The investigating physicians were not blinded to patient group allocation.

### 2.5. Statistical Methods

The Shapiro–Wilk test was used test for normal distribution. Chi-squared tests were used to analyze categorial data, and t-tests were used to analyze numerical data for group comparisons at the time of examination (T0 and T1). If the data were not normally distributed, the Mann–Whitney U test was used to analyze unpaired samples with ordinal variables. Data were analyzed using the SPSS statistical software package (IBM, Armonk, NY, USA), version 26.0, for Windows. The analysis was performed according to the protocol to account for attrition due to the pandemic situation.

## 3. Results

Of the 70 participants who completed the study between May 2020 and February 2021, the mean age was 48.4 years (SD 13), with 61.4% being female (see Table 1). Figure 1 shows the dropout rate and reasons for dropout. The t-tests at the baseline showed no significant differences between the IG and CG in terms of age, sex, tinnitus duration, characteristics or localization, pain, or range of motion (see Table 1). The IG (*n* = 37) received an average of 17.24 manual therapy sessions, ranging from a minimum of 13 to a maximum of 18 sessions, each lasting between 20 and 30 min. These sessions were delivered over a period of between 9 and 28 weeks. Any harm, such as bruising or worsening of symptoms, was documented in patient files. No adverse events or harm were reported in the study.

### 3.1. Modulation of Tinnitus and Dizziness

At the baseline, the CG showed a significantly more frequent modulation of the right (Phi = 0.253; *p* = 0.035) and left (Phi = 0.339; *p* = 0.005) levator scapulae muscles than the IG. This was also evident at T1, with a significantly greater modulation of tinnitus and/or dizziness via the levator scapulae muscle on the right (Phi = 0.428; *p* < 0.001) and on the left (Phi = 0.428; *p* < 0.001). Physical examinations revealed significant differences between the groups in 14 out of 18 muscles at T1 following the intervention. The IG showed significantly less modulation than the CG (see Table 2).

### 3.2. Pressure Pain of Head and Neck Muscles

At the baseline, the CG had significantly more frequent pressure pain of the masseter muscle on the right side than the IG (Phi = 0.252; *p* = 0.035) at the baseline, and this difference was also evident at T1. Muscle examination at T1 revealed significant differences in pressure pain in 10 out of 18 muscles. After the intervention, the IG had a significantly lower frequency of pressure pain in the muscles examined than the CG (see Table 3).

### 3.3. Cervical Spine Mobility

At the baseline, there was no significant difference in cervical spine mobility between the IG and the CG. At T1, however, the IG exhibited significantly higher mean values of rotation to the right (U = 343.00; *p* = 0.001) and left (U = 395.50; *p* = 0.008) than the CG (see Table 4).

### 3.4. Experiencing Tinnitus

**Figure 2:**
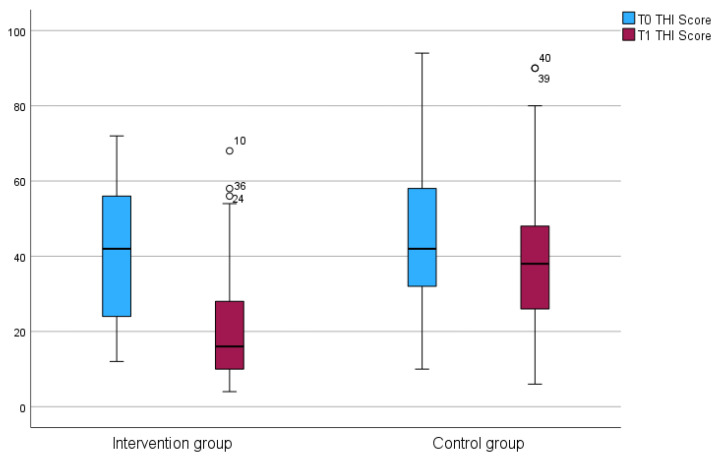
Boxplots of THI differences between the IG and CG at T0 and T1.

According to the Shapiro–Wilk test, the THI data were not normally distributed. At the baseline, Mann–Whitney U test showed that there was no significant difference in the distribution of THI severity levels between the IG and CG (U = 678.5; *p* = 0.423). At T1, however, there was a significant difference in the distribution of severity levels between the two groups (U = 944.0; *p* < 0.001) (Figure 2). A 17-point reduction, representing a strong improvement, was achieved by 48.65% (*n* = 18) in the IG and by 6.06% (*n* = 2) in the CG. This shows a significant difference between the groups (U = 22.000; z = −4.520; *p* < 0.001; *n* = 70) (see Table 5 and Figure 2).

### 3.5. Experiencing Dizziness

**Figure 3:**
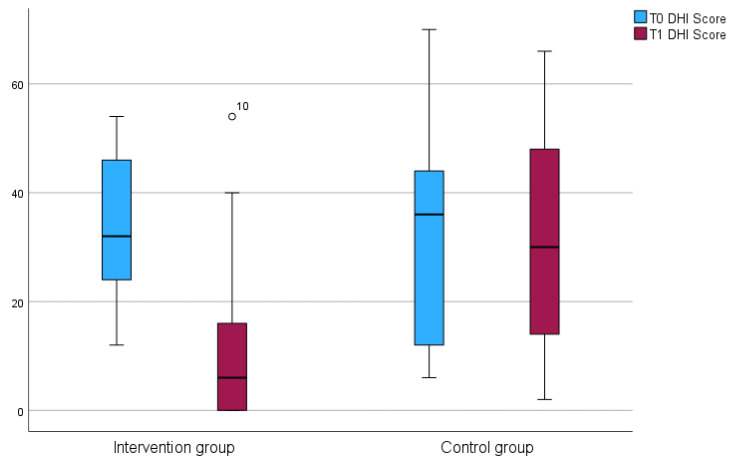
Boxplots of DHI differences between the IG and CG at T0 and T1.

**Figure 4:**
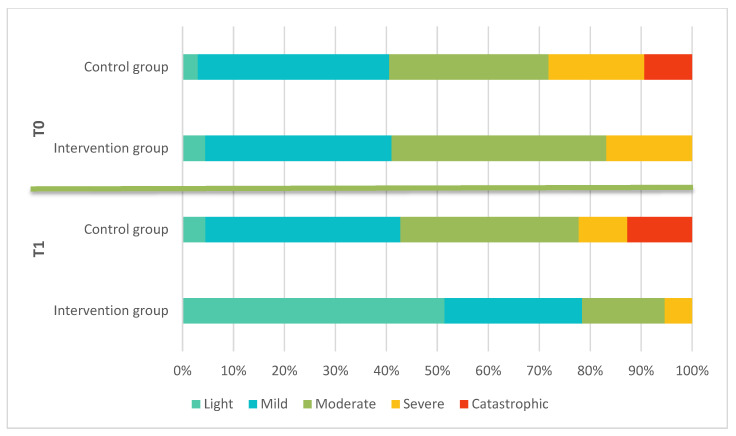
THI group affiliations for the intervention group and the control group at T0 and T1 in %.

The DHI data were not normally distributed, as confirmed by the Shapiro–Wilk test (*p* < 0.05). At the baseline, the Mann–Whitney U test showed that the groups were not significantly different in terms of mean scores and DHI categories (U = 73.5; *p* = 0.579) (Figure 3). At T1, however, the medians of the IG (M*d* = *6*) and CG (M = 30) were significantly different (*p* < 0.001; r = 0.47). In the IG, the DHI scores improved, demonstrating a clinically relevant change according to Kurre [23] (Md*^IG T^*^0^ = 32; Md*^IG T^*^1^ = 6) (see Table 5 and Figure 4).

## 4. Discussion

Manual therapy reduced tinnitus modulation and pressure pain, improving the range of motion of the cervical spine. It also significantly improved tinnitus- and dizziness-related disability in the IG compared with the CG.

The examination revealed irregularities in the neck muscles, particularly the cervical extensors, the trapezius muscle, and the levator scapulae muscle, in our sample of patients with CST. These irregularities have been reported less frequently in previous studies [31]. The results for the splenius capitis muscle and the semispinalis capitis muscle are particularly significant. Internationally, only one clinical investigation has been conducted on the splenius capitis muscle [32]. No research has been conducted on the semispinalis capitis muscle. Further research is required.

The improvement in range of motion (see Table 4) may have been due to manual therapy significantly reducing muscle hypertonia in the splenius capitis, semispinalis capitis, and trapezius pars descendens muscles. These muscles are partly responsible for the rotating of the skull. When these muscles are tense, the resulting shortening restricts rotation to the opposite side.

Manual therapy resulted in significant improvements in THI compared with the control group. Of the 37 patients in the IG, 12 showed a relevant improvement, while 18 showed a strong improvement. Figure 2 shows that 30 out of 37 patients significantly benefited from manual therapy. These results are consistent with those of Michiels et al. [28]. As tinnitus distress is negatively correlated with quality of life [7,8,9], manual therapy appears to impact on quality of life by improving THI values. However, this needs to be investigated further in additional studies.

The DHI also showed a significant improvement in reported dizziness scores (see Table 5). This improvement was evident in nine out of thirteen patients from the IG who experienced dizziness alongside tinnitus. Eight of these patients showed a strong improvement. Cheng et al. found that DHI values also correlate highly with health-related quality of life as well as anxiety and depression [33]. Therefore, it can be assumed that the clinical improvements in DHI were associated with improvements in quality of life and anxiety. However, this needs to be investigated further in future studies.

Concurrent changes in pressure pain of the cervicocranial muscles, an improved range of motion, and the THI results suggest that there is a relationship between the neck extensors and tinnitus symptoms. This is also supported by other evidence. For example, Michiels et al. showed that CST patients were more likely to have cervical spine dysfunction than patients without CST [34]. This is consistent with the theory of stimulus overload at the cranial nerve nuclei. The large amount of sensory information from tense neck muscles leads to cross-circuitry with the vestibular nucleus. However, it is not currently possible to establish a clear physiological causality. The clinical picture observed in the manual examination and the results of this study are consistent with the brainstem irritation model [15,16]. This shows that these are not independent symptoms. Rather, they are symptoms that can be triggered by a functional state of brainstem irritation.

The study was conducted during the pandemic, which is one of its limitations. It is not possible to assess whether this had a positive or negative influence. However, it is clear that patients dropping out of the study due to SARS-CoV-2 infection negatively impacted the study. Furthermore, the additional training required for manual therapy was the only aspect of the therapeutic techniques that was pre-specified. Future studies should examine if different techniques have different effects on the muscles, how many treatments are effective, and how sustainable the intervention is. Further studies should also examine the role of individual muscles. For example, one group of patients could undergo treatment in the area of the neck extensors only, while another group could undergo treatment in the areas of the trapezius pars descendens and levator scapulae muscles only, or in other areas such as the masticatory muscles. This trial used a waitlist design. Further trials could compare an intervention group with a control group receiving usual physiotherapy care. Each assessment and examination was carried out by the same physician. Although this carries a risk of bias, it also ensures consistency in the professional assessment. Further objectification could be achieved by using a pressure pain measuring device (“Tissue Tension Meter”). As this study did not distinguish between primary and secondary tinnitus, it cannot be ruled out that this may have influenced the results. Therefore, future studies should include a full tinnitus diagnosis.

## 5. Conclusions

The study shows that manual therapy can effectively treat myofascial disorders of the head and neck and reduce tinnitus- and dizziness-related distress in a clinically significant way. In order to optimize the treatment of patients with CST, examination and treatment could be more targeted towards the muscles identified in the study.

## Figures and Tables

**Table 1:** Tinnitus characteristics of participants at T0 (*n*: frequency; SD: standard deviation).

Category		Values	*p*
Age, mean (SD)		48 (13)	0.239
Sex (female, *n* (%))		43 (61.4)	0.091
Tinnitus duration, *n* (%)			0.353
	<3 months	7 (10)	
	<6 months	8 (11.4)	
	<12 months	9 (12.9)	
	>2 years	14 (20)	
	>5 years	11 (15.7)	
	>10 years	21 (30)	
Sound, *n* (%)	Whistling/beeping	57 (81.4)	0.131
	Humming	18 (25.7)	0.207
	Knocking	1 (1.4)	0.162
	Other	11 (15.7)	0.299
Localization, *n* (%)			0.116
	Right ear	8 (11.4)	
	Left ear	19 (27.1)	
	On both sides	43 (61.4)	
THI median (IQR)		47 (35)	0.145
DHI median (IQR)		34 (25)	0.363

**Table 2:** Frequencies of tinnitus modulation and dizziness by muscle palpation for the IG and CG at T0 and T1 and results of the chi-squared test to examine group differences at T0 and T1 (IG: intervention group; CG: control group; *n*: number of participants; Phi: measure of association based on the chi-squared statistic; *p*-value: probability. * significant difference between the two groups (*p* ≤ 0.05)).

	T0				T1			
Muscles, *n* (%)	CG Frequence *n* = 33	IG Frequence *n* = 37	Phi	*p*	CG Frequence *n* = 33	IG Frequence *n* = 37	Phi	*p*
Spleniuscapitis, right	24 (72.7)	25 (67.6)	0.056	0.638	26 (78.8)	11 (29.7)	0.491	<0.001 *
Spleniuscapitis, left	31 (93.9)	32 (86.5)	0.124	0.299	27 (81.8)	18 (48.7)	0.346	0.004 *
Semispinaliscapitis, right	21 (63.6)	24 (64.9)	−0.013	0.915	23 (69.7)	11 (29.7)	0.399	0.001 *
Semispinaliscapitis, left	29 (87.9)	31 (83.8)	0.058	0.625	27 (81.8)	17 (45.9)	0.371	0.002 *
Temporalis, right	6 (18.2)	7 (18.9)	−0.009	0.937	8 (24.2)	4 (10.8)	0.178	0.137
Temporalis, left	6 (18.2)	4 (10.8)	0.105	0.379	10 (30.3)	3 (8.1)	0.285	0.017 *
Masseter, right	16 (48.5)	10 (27.0)	0.222	0.064	13 (39.4)	10 (27.0)	0.131	0.271
Masseter, left	21 (63.6)	22 (59.5)	0.043	0.720	17 (51.5)	10 (27.0)	0.251	0.036 *
Pterygoideus, right	11 (33.4)	10 (27.0)	0.069	0.565	13 (39.4)	6 (16.2)	0.260	0.029 *
Pterygoideus, left	14 (42.4)	10 (27.0)	0.162	0.175	17 (51.5)	9 (24.3)	0.281	0.019 *
Floor-of-mouth muscles, right	9 (27.3)	10 (27.0)	0.003	0.982	17 (51.5)	10 (27.0)	0.251	0.036 *
Floor-of-mouth muscles, left	11 (33.4)	10 (27.0)	0.069	0.565	20 (60.6)	10 (27.0)	0.339	0.005 *
Trapezius, right	18 (54.6)	14 (37.8)	0.167	0.161	20 (60.6)	8 (21.6)	0.397	0.001 *
Trapezius, left	14 (42.4)	14 (37.8)	0.047	0.696	24 (72.7)	7 (18.9)	0.541	<0.001 *
Levator scapulae, right	19 (57.6)	12 (32.4)	0.253	0.035 *	20 (60.6)	7 (18.9)	0.428	<0.001 *
Levator scapulae, left	20 (60.6)	10 (27.0)	0.339	0.005 *	20 (60.6)	7 (18.9)	0.428	<0.001 *
Sternocleidomastoideus, right	7 (21.2)	6 (16.2)	0.064	0.592	6 (18.2)	3 (8.1)	0.150	0.209
Sternocleidomastoideus, left	7 (21.2)	8 (21.6)	−0.005	0.967	9 (27.3)	6 (16.2)	0.135	0.260

**Table 3:** Frequencies of pressure pain of muscles for the IG and CG at T0 and T1 and results of the chi-squared test to examine group differences at T0 and T1 (IG: intervention group; CG: control group; *n*: number of participants; Phi: measure of association based on the chi-squared statistic; *p*: probability. * significant difference between the two groups).

	T0				T1			
Muscles, *n* (%)	CG Frequence *n* = 33	IG Frequence *n* = 37	Phi	*p*	CG Frequence *n* = 33	IG Frequence *n* = 37	Phi	*p*
Spleniuscapitis, right	31 (93.9)	37 (100)	−0.182	0.129	32 (96.9)	27 (73.0)	0.329	0.006 *
Spleniuscapitis, left	31 (93.9)	37 (100)	−0.182	0.129	31 (93.9)	31 (83.8)	0.159	0.182
Semispinaliscapitis, right	31 (93.9)	33 (89.2)	0.085	0.479	32 (96.9)	25 (67.6)	0.377	0.002 *
Semispinaliscapitis, left	30 (90.9)	37 (100)	−0.224	0.061	33 (100)	26 (70.3)	0.408	0.001 *
Temporalis, right	11 (33.4)	9 (24.3)	0.100	0.405	19 (57.6)	13 (35.1)	0.225	0.060
Temporalis, left	9 (27.3)	9 (24.3)	0.034	0.778	16 (48.5)	17 (46.0)	0.025	0.832
Masseter, right	25 (75.8)	19 (51.4)	0.252	0.035 *	28 (84.9)	23 (62.2)	0.255	0.033 *
Masseter, left	25 (75.8)	28 (75.7)	0.001	0.994	29 (87.9)	29 (78.4)	0.126	0.292
Pterygoideus, right	18 (54.6)	19 (51.4)	0.032	0.789	26 (78.8)	22 (59.5)	0.208	0.082
Pterygoideus, left	23 (69.7)	20 (54.1)	0.160	0.180	23 (69.7)	21 (56.8)	0.134	0.263
Floor-of-mouth muscles, right	13 (39.4)	14 (37.8)	0.016	0.894	25 (75.8)	12 (32.4)	0.433	<0.001 *
Floor-of-mouth muscles, left	17 (51.5)	12 (32.4)	0.193	0.106	20 (60.6)	9 (24.3)	0.368	0.002 *
Trapezius, right	29 (87.9)	35 (94.6)	−0.120	0.316	32 (96.9)	30 (81.1)	0.249	0.037 *
Trapezius, left	30 (90.9)	34 (91.9)	−0.018	0.883	32 (96.9)	26 (70.3)	0.354	0.003 *
Levator scapulae, right	28 (84.9)	31 (83.8)	0.015	0.903	31 (93.9)	26 (70.3)	0.304	0.011 *
Levator scapulae, left	31 (93.9)	32 (86.5)	0.124	0.299	29 (87.9)	21 (56.8)	0.344	0.004 *
Sternocleidomastoideus, right	9 (27.3)	7 (18.9)	0.099	0.406	8 (24.2)	8 (21,6)	0.031	0.794
Sternocleidomastoideus, left	4 (12.1)	5 (13.5)	−0.021	0.862	8 (24.2)	8 (21.6)	0.031	0.794

**Table 4:** Cervical spine mobility of the IG and CG at T0 and T1 (IG: intervention group; CG: control group; *n*: number of participants; U: Mann–Whitney U test; *p*: probability. * significant difference between the two groups).

T0, *n* (%)	IG Median (IQR) *n* = 37	CG Median (IQR) *n* = 33	U	*p*
Inclination	55 (15)	55 (13)	569.00	0.622
Reclination	60 (20)	60 (20)	610.00	0.995
Lateral inclination, right	35 (15)	35 (10)	583.00	0.742
Lateral inclination, left	35 (15)	35 (13)	556.50	0.518
Rotation, right	60 (8)	60 (10)	565.50	0.577
Rotation, left	60 (10)	60 (13)	534.00	0.351
T1, *n* (%)				
Inclination	50 (25)	55 (18)	556.00	0.518
Reclination	65 (20)	55 (18)	516.50	0.265
Lateral inclination, right	40 (15)	35 (10)	467.50	0.087
Lateral inclination, left	40 (15)	35 (10)	466.00	0.084
Rotation, right	60 (10)	60 (10)	343.00	0.001 *
Rotation, left	60 (10)	60 (10)	395.50	0.008 *

**Table 5:** Differences in clinical changes from T0 to T1 in THI and DHI total values using Mann–Whitney U test (THI: Tinnitus Handicap Inventory; DHI: Dizziness Handicap Inventory; *n*: number of participants; Phi: measure of association based on the chi-squared statistic; *p*: probability. * significant difference between the two groups).

	THI		DHI	
	Mann–Whitney U = 227.000; z = −4.520; *p* < 0.001 *; *n* = 70		Mann–Whitney U = 29.500; z = −2.826; *p* = 0.003 *; *n* = 26	
	Intervention Group (*n* = 37)	Control Group (*n* = 33)	Intervention Group (*n* = 13)	Control Group (*n* = 13)
Relevant improvement, *n* (%)	12 (32.4)	7 (21.2) (7)	1 (7.7)	3 (23.1)
Strong improvement, *n* (%)	18 (48.7)	2 (6.1)	8 (61.5)	0 (0)

## Data Availability

Due to data privacy rules and according to German law (§ 75 SGB X), access to the data is granted only to responsible scientific personnel at Hannover Medical School, Hannover, Germany, within the framework of the respective research project. It is not permitted to give third parties access to the data without a research proposal approved by the principal investigator. The trial protocol and statistical analysis plan can be accessed via the corresponding author.

## Abbreviations

The following abbreviations are used in this manuscript:

THI	Tinnitus Handicap Inventory Questionnaire
DHI	Dizziness Handicap Inventory
CST	Cervicogenic somatosensory tinnitus
